# Research on the Application of Visual Recognition in the Engine Room of Intelligent Ships

**DOI:** 10.3390/s22197261

**Published:** 2022-09-25

**Authors:** Di Shang, Jundong Zhang, Kunxin Zhou, Tianjian Wang, Jiahao Qi

**Affiliations:** 1 College of Marine Engineering, Dalian Maritime University, Dalian 116026, China; 2 China Classification Society Dalian Branch, Dalian 116001, China

**Keywords:** intelligent ship, visual recognition, YOLOv5, object detection, model improvement

## Abstract

In the engine room of intelligent ships, visual recognition is an essential technical precondition for automatic inspection. At present, the problems of visual recognition in marine engine rooms include missing detection, low accuracy, slow speed, and imperfect datasets. For these problems, this paper proposes a marine engine room equipment recognition model based on the improved You Only Look Once v5 (YOLOv5) algorithm. The channel pruning method based on batch normalization (BN) layer weight value is used to improve the recognition speed. The complete intersection over union (CIoU) loss function and hard-swish activation function are used to enhance detection accuracy. Meanwhile, soft-NMS is used as the non-maximum suppression (NMS) method to reduce the false rate and missed detection rate. Then, the main equipment in the marine engine room (MEMER) dataset is built. Finally, comparative experiments and ablation experiments are carried out on the MEMER dataset to verify the strategy’s efficacy on the model performance boost. Specifically, this model can accurately detect 100.00% of diesel engines, 95.91% of pumps, 94.29% of coolers, 98.54% of oil separators, 64.21% of meters, 60.23% of reservoirs, and 75.32% of valves in the actual marine engine room.

## 1. Introduction

In recent years, with the development of vision technology and other related technologies, it has been thought that future shipping vessels are bound to develop in the direction of intelligence. An intelligent engine room is an integral part of intelligent ships. However, there are many scientific problems associated with the realization of an intelligent engine room, such as situation awareness, path planning, target detection, automatic discrimination [1,2], etc. Therefore, it is important to explore the application of many critical technologies in the engine room of intelligent ships. The development of marine engine room monitoring is a process in which automation and intelligence gradually replace human labor. At the same time, visual recognition technology will gradually replace the naked eye.

Currently, marine engine room automation is mainly accomplished by the engine room monitoring and alarm system, supplemented by the inspection of the engineer on duty. However, engine room inspection is an indispensable task to ensure the safe navigation of the ship, because the monitoring and alarm system can only directly identify the specific fault of specific equipment but cannot further show the deeper fault that leads to the alarm or carry out early fault prediction. For example, when the temperature of the high-temperature freshwater of the main engine is higher than the upper limit, the monitoring and alarm system will issue an alarm. However, the specific fault causes still need to be eliminated by the engineer on duty one by one. If the alarm of pipeline system leakage is caused by long-term seawater corrosion, then the engineer can discover the fault in advance by the change in the appearance of the pipeline during inspection, to avoid such an alarm. Furthermore, if a certain number of visual sensors can be arranged in the marine engine room to cover the main equipment and monitor the real-time running state and appearance, it can prevent this kind of failure or defects in advance, eliminate hidden danger as soon as possible, reduce the probability of failure and ensure the safety of ship sailing. At the same time, it can provide technical support for the maintenance and decision-making of intelligent engine rooms, which has far-reaching significance. However, there is still insufficient research on the application of visual recognition in marine engine rooms, and current research still faces some difficulties and challenges. In summary, it mainly includes the following aspects:

- There are no public datasets for the main equipment of marine engine rooms, and there is a lack of relevant research material and exact data. In the field of marine engine room visual recognition, there are no known reliable open sources datasets at present, and the very few existing works of literature on marine industry equipment detection are of little reference significance.
- There is a wide variety and dense layout of equipment in a marine engine room. The size of adjacent equipment may vary by several orders of magnitude. For example, there are large-scale differences between the main engine and valve, reservoir and meter, which increases the difficulty of detection and recognition.
- The equipment in marine engine rooms is densely arranged, and the pipelines are staggered and complicated. Due to the compact layout and the pipelines’ connection characteristics of marine engine rooms, there is widespread occlusion or overlap among various equipment.

In the face of the above problems, this paper proposes a marine engine room equipment identification algorithm based on improved YOLOv5. The specific work is as follows:

- A MEMER dataset is built, relying on the resources of Dalian Maritime University’s three-dimension virtual marine engine room project team. We built the MEMER dataset by processing photos taken in actual engine rooms. The ship types of the dataset include a very large crude oil carrier (VLCC), very large container ship (VLCS), and very large ore carrier (VLOC), and the equipment category includes diesel engines, pumps, coolers, oil separators, meters, reservoirs, and valves. The details of data processing will be shown in Section 4.1.
- The channel pruning based on the BN layer [3] weight value is used to accelerate the recognition speed. To improve the accuracy of recognition in complicated engine rooms, the CIoU_Loss loss function and hard-swish activation function are used to optimize the original algorithm. Meanwhile, the soft-NMS is used as the NMS method to reduce the false rate and missed rate of detection.

The arrangement of the remainder is as follows: the second part discusses the related work on visual recognition; the third part explicitly introduces the detection model of marine engine room equipment based on improved YOLOv5; the fourth part carries out experiments and verification based on the MEMER dataset; the fifth part summarizes and prospects the research contents.

## 2. Preliminary

Visual recognition is a prerequisite for scene perception and other visual tasks. Due to the development of computer technology, this field has been widely explored and studied. Specifically, visual recognition needs to provide basic information about objective objects in a digital image, such as the location of a human, animal, or car. It is also an essential part of many other tasks, such as semantic segmentation [4], object tracking [5], and scene description [6]. In recent years, significant breakthroughs have been made In marine and ocean engineering, such as automatic ship identification systems [7], detection of alien species in ports [8], undersea exploration [9], marine farming, and fishing [10], etc.

The development of object detection can be divided into the following two stages: the traditional object detection period and the object detection period based on deep learning (DL). Image processing is a basic mainstream method in the traditional object detection period. It is successively divided into candidate box selection, feature extraction, classification and optimization result [11,12]. In the period of the target detection based on DL, the algorithm can be roughly divided into two categories, anchor-based and anchor-free [13,14]. The former can be divided into two-stage and one-stage algorithms. The two-stage detection algorithm is a step-by-step process based on candidate regions, with high accuracy and slow speed. The single-stage detection algorithm is based on bounding box regression. The detection network generates candidate boxes and performs classification and regression simultaneously, with high speed and low accuracy [15]. Although the anchor-based detection model has an excellent performance in speed and accuracy, most preset anchor frames are negative samples, which will aggravate the imbalance of positive and negative samples in the training process. The preset anchor frame is artificially designed, and its width-to-height ratio is sensitive to the dataset, affecting the detection performance.

**Figure 1:**
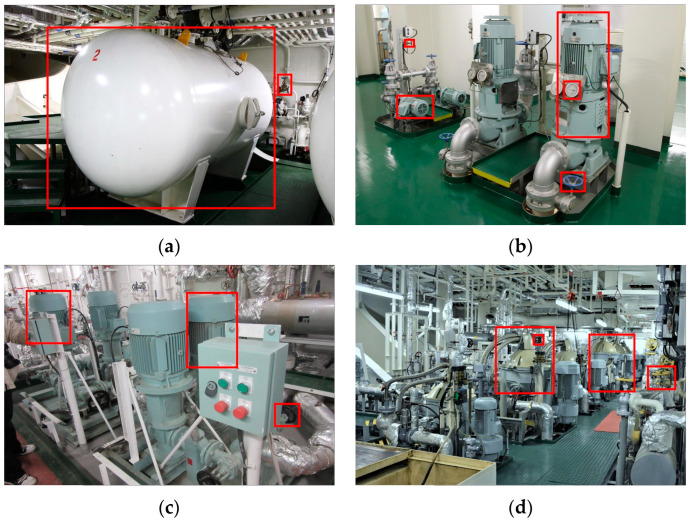
The difficulties of visual recognition in a marine engine room. In (**a**), the reservoir takes up more than 55% of the image while the valve accounts for less than 1%. In (**b**), the scale differences between the same equipment are general. In (**c**), the pumps and the valve are occluded by the former equipment. In (**d**), the overlaps between the equipment will cause an impact on the training process.

The spatial structure of marine engine rooms is complex, the scale gap between different equipment is vast, and the apparent similarity of the same equipment is very low. As shown in Figure 1a, the reservoir accounts for more than 55% of the pixels in the whole image, which is a large target, while the valve accounts for less than 1% of the pixels, which is a small target. They are not only very different in scale, but also very different in shape. Similar situations are also reflected in Figure 1b. For example, there are scale differences between pump and pump, pump and meter, pump and valve, valve and valve, which bring difficulties to the detection of marine engine room equipment. Due to the compact layout and the pipelines’ connection characteristics of marine engine rooms, there is a widespread phenomenon of occlusion or overlap among various equipment. IN addition, there is overlap between the meter and valve, as shown in Figure 1b below. In Figure 1c, the pump local control box partially shields the pump body and valve parts. In Figure 1d, there are overlaps between the separator and valve parts, which will cause interference in prediction box regression in model training.

In response to the above problems, an improved auxiliary engine detection algorithm based on a single shot multibox detector (SSD) was proposed in [16] by adding repulsion loss for the overlapping targets to improve the detection effect of the model against dense occlusion and overlap. Ref. [17] introduced the single channel plain architecture (RepVGG) as the feature extraction network of the basic framework RetinaNet to simplify the complexity and improve real-time detection. Meanwhile, the neighbor erasing and transferring mechanism was applied in the feature pyramid to deal with complicated scale variations. This method demonstrates particular improvements in accuracy and real-time performance of recognition. It also considers the situation of complicated scale variations. However, these detection methods cannot solve the problem of missing detection and can only deal with a specific part of the problems. They are not very suitable for application in actual marine engine rooms.

The YOLOv5 algorithm adopted in this paper is already the fifth generation of the YOLO series, which has been widely studied by researchers and has excellent performance in visual recognition and defect detection. Ref. [18] proposed TPH-YOLOv5 for drone-captured scenarios, in which one prediction head is used to detect different-scale objects, while the other is a transformer prediction head (TPH) to explore the prediction potential with a self-attention mechanism. The convolutional block attention model (CBAM) was also integrated to find attention regions in dense objects scenarios. In the VisDrone Challenge 2021, the TPH-YOLOv5 won fifth place. Compared to the baseline model (YOLOv5), the TPH-YOLOv5 improves by about 7%. Ref. [19] presented a defects detector on a steel surface, based on improved YOLOv5, named MSFT-YOLO. The TRANS module designed based on a transformer was added to the backbone and detection headers to combine the features and global information. The fusion of features at different scales by combining multi-scale feature fusion structures enhanced the dynamic adjustment of the detector to objects at different scales. In brief, it is effective at great image background interference, confused defect categories, and complicated scale variations in defects in industrial scenes. Finally, the performance boost of the MSFT-YOLO compared with baseline YOLOv5 was validated on the NEU-DET dataset. It is about 7% higher than the baseline model (YOLOv5) and 18% higher than Faster R-CNN (convolutional neural networks). Ref. [20] proposed a ship detection algorithm based on improved YOLOv5 to improve ship detection accuracy and real-time performance. The feature extraction process was merged with the GhostbottleNet algorithm to overcome the incomprehensive feature capture problem in the original YOLOv5 network, due to the inhomogeneous distribution of ship image features in transverse and vertical. Finally, experimental verification shows that the mAP of the improved network is 99.85%, which is 2.18% higher than the original. In addition, compared with the baseline network, the mAP of the improved network can reach the highest value in a short time, and the range of change is smaller than the baseline.

According to the above research and existing problems in marine engine room equipment detection, this paper proposes an improved measure based on the YOLOv5 algorithm. The specific schemes are presented in the next section.

## 3. Amelioration

The size of the YOLOv5 model is 90.1 MB [21]. Although compared with previous versions of the YOLO model, it has been reduced, it still involves a lengthy training process and has poor real-time detection performance. In this paper, first of all, from the perspective of the network structure, the channel pruning strategy is used to diminish the model to improve the detection speed. Secondly, the loss function and activation function improvement aim at enhancing the detection accuracy and missed detection rate.

### 3.1. Model Principle

**Figure 2:**
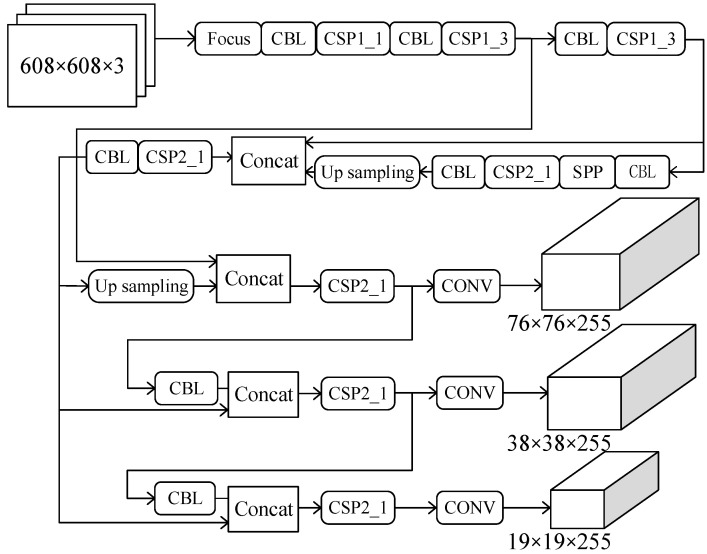
YOLOv5 network structure diagram.

YOLOv5 is the latest version of the YOLO series, which has launched four models of different depths. This paper adopts YOLOv5l [22], with deeper network layers as the research object. Figure 2 shows the network structure of YOLOv5.

#### 3.1.1. Input

YOLOv5 uses adaptive anchor box calculations and adaptive picture scaling as the input. The size of the anchor box needs to be set in advance before training the object detection algorithm. During the training, the prediction box and the real box can be matched according to the set size of the anchor box, and then the difference can be calculated and propagated back. In the previous version of the YOLO algorithm, the anchor box calculation is implemented by a separate program. At the same time, YOLOv5 integrates the anchor box calculation with the algorithm model to adaptively calculate the anchor box size, according to the dataset’s characteristics in the training process, which improves the model training efficiency.

When collecting data, to obtain various types of images, they are usually taken at different angles, so the size of the collected images is different. However, YOLOv5 requires the input of images of a uniform size. To solve this problem, YOLOv5 uses adaptive image scaling. The input image size set in this paper is 640 × 640. YOLOv5 will retain the original aspect ratio to scale the image and fill both ends with grey edges, once the input image is inconsistent. For example, when the input image size is 960 × 720, the scaling ratio must be calculated first. The scaling ratio of length and width is 0.67 and 0.89, respectively. The smaller value is selected to scale the image, and the scaled image size is 640 × 480, then both ends of the short side are filled with grey edges of 80 width, respectively, and the final 640 × 640 picture is accomplished. This filling method is only used in training; when testing, YOLOv5 will fill the image with the smallest grey edge adaptively to improve the reasoning speed of the model.

#### 3.1.2. Backbone

**Figure 3:**
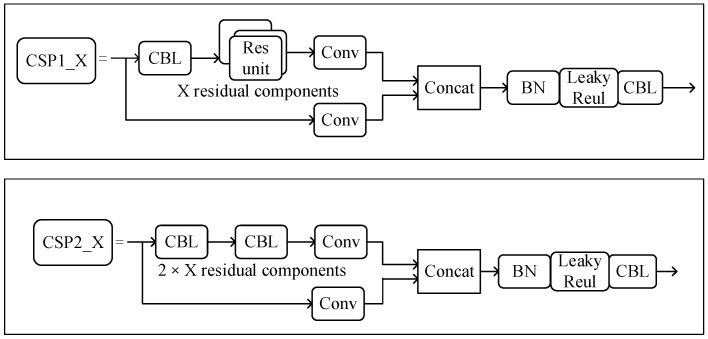
The structure diagram of CSP1_X and CSP2_X.

YOLOv5 adopts the cross stage partial (CSP) [23] net structure on Backbone. The CSP structure is shown in Figure 3. It uses the split-merge strategy to divide the feature map into two parts to add gradient paths and to allow the gradient information to spread from different paths, then merge through splicing, which can solve the problem of gradient information duplication in network propagation to reduce the computation and enhance the learning ability of the network.

**Figure 4:**
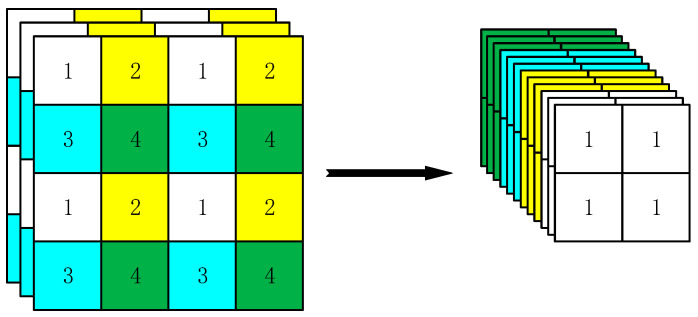
Slicing operation.

YOLOv5 uses the FOCUS module for Backbone for the first time. The FOCUS module first slices the picture and turns one into four complementary pictures, and each picture saves different information. The input channel is expanded four times and then carries out down-sampling through convolution operation. The slicing operation is shown in Figure 4. The function of the FOCUS module is not to cause information loss when the image is down-sampled, which makes the feature extraction more adequate. The drawback is that computation increases.

**Figure 5:**
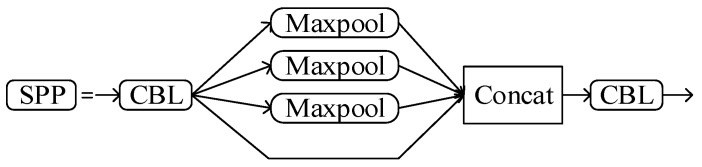
Schematic diagram of SPP.

Spatial pyramid pooling (SPP) [24] is a feature fusion structure, whose function is to transform inputs of different sizes into outputs of the same size. The structure of SPP is shown in Figure 5. First, we carry out the basic convolution operation. Then, pooling operations with different sizes of convolution kernels are carried out, and filling is performed before pooling to ensure that the size of feature maps before and after pooling remain unchanged. Finally, feature maps with different sizes are spliced to achieve feature fusion.

#### 3.1.3. Neck Structure

**Figure 6:**
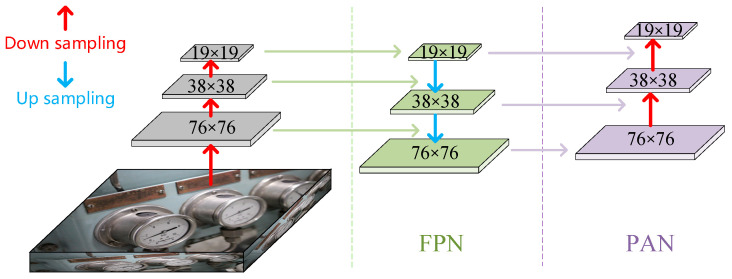
Schematic diagram of FPN + PAN.

The neck structure of YOLOv5 combines feature pyramid networks (FPN) [15] and path aggregation networks (PAN) [25], which is a part of the features fusion. The FPN+PAN structure is shown in Figure 6. FPN transmits feature information from top to bottom, transfers deep feature information to shallow layers, and enhances the expression ability of features at different scales. PAN transmits feature information downwards, transferring the position information from the shallow layer to the deep layer, enhancing the positioning ability of features at different scales to realize the fusion of different features.

#### 3.1.4. Output

The output of YOLOv5 consists of the generalized intersection over union (GIoU) loss function and weighted NMS. The GIoU_Loss loss function was proposed in 2019 [26]. Its appearance solves the problem that intersection over union (IoU) loss cannot identify the alignment in different ways, and the function is not differentiable when the prediction box and the real box are disjointed.

**Figure 7:**
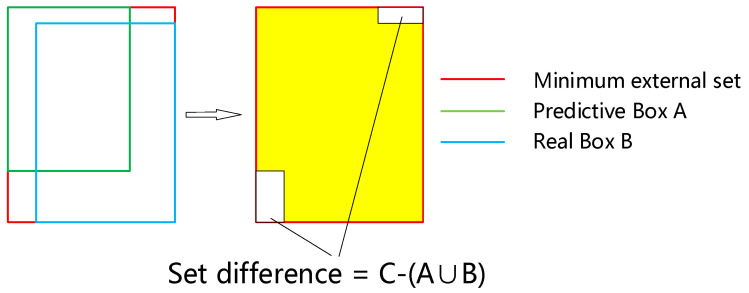
Schematic diagram of GIoU_Loss loss function.

As shown in Figure 7, GIoU_Loss first obtains the minimum external set C that can ultimately include prediction box A and real box B, then calculates the proportion of the area in C, except for A and B. The specific calculation formula is as follows:

(1) $$GIoU_{LOSS}=1-IOU+\frac{C-(A\cup B)}{C}$$

Compared with the *IoU_Loss* loss function, the *GIoU_Loss* loss function also considers the non-coincident region of the prediction box and the real box when calculating the values, which makes the calculation of the coincident degree of the two boxes more practical and reasonable.

NMS is mainly used to screen candidate boxes in the algorithm, which is an essential post-processing method in object detection. YOLOv5 uses weighted NMS as the NMS method. When screening candidate boxes, the weighted NMS does not directly delete the candidate boxes, whose IOU value is greater than the threshold, but linearly weights the candidate boxes, whose IOU value is greater than the threshold to obtain the final prediction box. The calculation formula is as follows:

(2) $$M=\frac{\sum_{i}\omega_{i}B_{i}}{\sum_{i}\omega_{i}},B_{i}\in \left\{B|IoU(M,B)\geq thresh\right\}\cup \left\{M\right\}$$

(3) $$\omega_{i}=s_{i}IoU(M,B_{i})$$

The $\omega_{i}$ is the weight of the *i*th candidate box, *B* indicates the set of candidate boxes whose IOU value is greater than the threshold, and the $s_{i}$ is the confidence of the *i*th prediction box.

### 3.2. YOLOv5 Improvement

With the development of the convolutional neural networks, the network level is increasingly deepened. Its volume and parameter number also gradually increase, so it becomes more and more difficult to deploy the model at the terminal directly. The current mainstream solution is to diminish the model, and the main ways include model pruning, model quantification and knowledge distillation [27].

#### 3.2.1. Channel Pruning Based on BN Layer Weight Value

Model pruning is one of the most influential and robust methods for compression. Model pruning mainly includes channel pruning and layer pruning. By analyzing the structure of the YOLOv5 model, this paper decided to adopt a channel pruning strategy based on the BN layer weight value to diminish the model [28].

**Figure 8:**
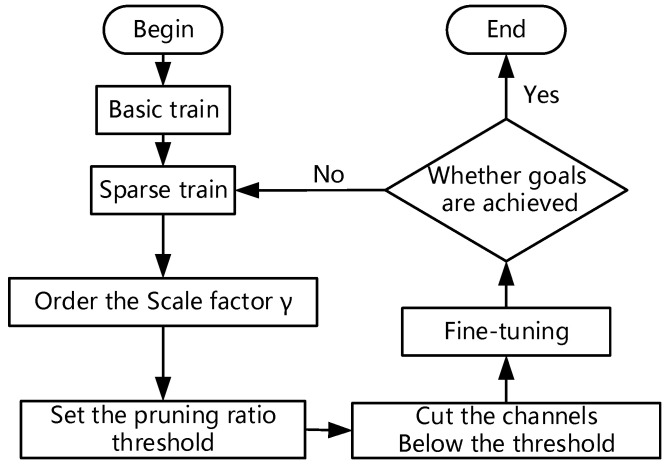
Flow diagram of model pruning.

The channel pruning strategy is shown in Figure 8. Firstly, basic training is carried out to make the model converge to higher precision. As a result of the depth of the model layer, the convolution calculation remains a large redundant number. Therefore, sparse training is required in the second step, and scaling factors are added to the BN layer. In the training process, the model will divide channels according to the importance of channels, and the weight of unimportant channels will be diminished. Then, the unimportant channels will be cut out, and the model’s accuracy will decrease. Finally, the accuracy will be restored by fine-tuning.

Sparse training is the most crucial step of model pruning. If sparse training is not sufficient, the accuracy of model pruning will be reduced to 0. Presently, the methods of sparse training mainly include weight sparse, layer sparse and channel sparse. Compared with the other two methods, channel sparsity has higher flexibility and realizability, so it is the best sparse method. Channel sparsity means that before entering the next layer of convolution, the output of each channel is multiplied by a scaling factor γ, and the network weight value is combined for training. The model will automatically learn to adjust the scaling factor γ in the training process, and the γ of the unimportant channel will gradually reach 0.

BN is a common optimization method in convolutional neural networks to improve the convergence speed of the model and solve the problem of gradient disappearance. Each convolutional layer of YOLOv5 has a BN layer, so the channel pruning strategy based on BN layer weight value can obtain a higher model compression rate, without incurring additional computation. The principle is as shown in Equation (4); $z_{in}$ and $z_{out}$ represent the input and output, $\mu_{B}$ and $\sigma_{B}$ are the mean value and variance in each batch and γ and β are the parameters of the scaling scale and displacement value. Channel sparsity based on the BN layer directly takes γ as the scaling factor [29], which has the advantage of not introducing new parameters into the network and not increasing the computational complexity and cost of the model.

(4) $$\hat{z}=\frac{z_{in}-\mu_{B}}{\sqrt{\sigma_{B}{}^{2}+\varepsilon }};z_{out}=\gamma \hat{z}+\beta$$

(5) $$L=\sum_{\left(x,y\right)}l(f(x,W),y)+\lambda \sum_{\gamma \in \Gamma }g(\gamma )$$

In Equation (5), the scaling factor γ is combined with the loss function, and the L1 regularization method is selected. The scaling factor is trained jointly with the loss function to realize the sparsification of the scaling factor. The (x,y) represent the input and output, respectively, W represents the weight parameter of the model, the first term represents the original training loss, g(γ) is the penalty term, and λ is the factor used to balance the two terms.

**Figure 9:**
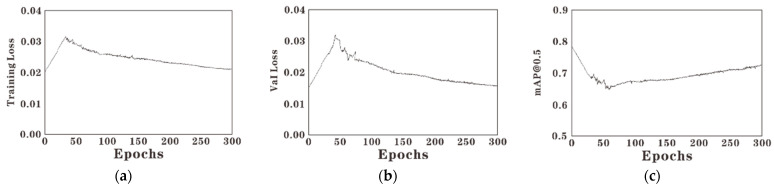
The graph of sparse training process. (**a**) is the training loss curve; (**b**) is the verified loss curve; (**c**) is the mAP curve.

In the process of sparse training, the size of scaling factor γ directly affects the training results. The larger γ is, the faster the model becomes sparse, but the faster the accuracy decreases. The smaller γ is, the slower the model becomes sparse, while the slower the accuracy decreases. In this paper, the value of γ is selected as 0.005 for sparse training, and the results are shown in Figure 9. The model accuracy curve shows a process of decreasing first and then increasing, and the final accuracy is lower than the initial value. In contrast, the training loss curve and validation loss curve are opposite.

**Figure 10:**
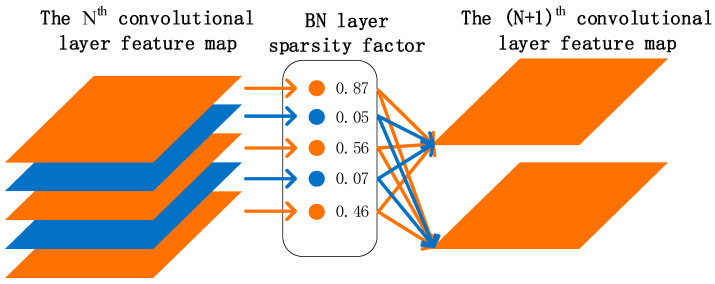
Schematic diagram of pruning.

After sparse training, a model with many γ values close to 0 is obtained. Channels with γ values close to 0 and corresponding weights are pruned by deleting all input and output. The principle is shown in Figure 10. Among all the channels in the Nth layer, there are two channels whose γ value is close to 0, and their contribution to the network model is low. Deleting them will not affect the network. After pruning the network layer by layer, the model volume will be compressed. A global threshold is artificially set during the pruning process to determine which channels need pruning.

#### 3.2.2. CIoU_Loss Loss Function

In this paper, CIoU_Loss [30] is used instead of GIoU_Loss as the loss function of the YOLOv5 model. When the prediction box and the real box have an inclusion relationship, its minimum external set *C* is the outer box, so the difference is 0. In this case, GIoU_Loss is equivalent to IoU_Loss, and only the size of the IOU value can be calculated. It cannot reflect the relative position of the two boxes. The calculation formula of the CIoU_Loss is as follows:

(6) $$CIoU_{LOSS}=1-IOU+\frac{\rho^{2}(b_{pred},b_{gt})}{C}+\alpha v$$

(7) $$\alpha =\frac{v}{(1-IOU)+v}$$

(8) $$v=\frac{4}{\pi^{2}}{\left[(\arctan\frac{w_{gt}}{h_{gt}})-\arctan\frac{w}{h}\right]}^{2}$$

α is the equilibrium proportion coefficient, $\rho^{2}(b_{pred},b_{gt})$ is the Euclidean distance between the center point of the prediction box $b_{pred}$ and the real box $b_{gt}$, c is the diagonal length of the minimum closed convex set, and v is used to measure whether the proportions of the two boxes are consistent. IOU is the intersection ratio of the two boxes.

**Figure 11:**
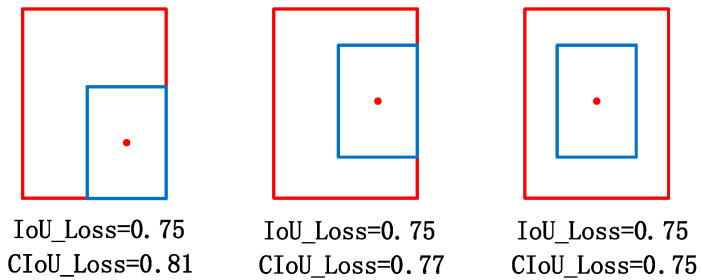
Description diagram of the relative position between the prediction box and the real box.

Compared with IoU_Loss, the CIoU_Loss loss function adds two penalty terms, among which the first term is the relative position relation penalty term of the two boxes. When the position relationship between the two boxes changes, the penalty term will change accordingly. When the two boxes contain each other, this method can also reflect the relative position of the outer box where the inner box is located, as shown in Figure 11. The second penalty term is the aspect ratio penalty, which measures whether the aspect ratio of the two boxes is consistent. The CIoU_Loss loss function not only considers the problem of the overlap rate, but also integrates the relative position relationship and the aspect ratio, which is more reasonable and will significantly improve the convergence speed of the model.

#### 3.2.3. Soft-NMS

Although the weighted NMS has a better candidate box screening effect and the model is more stable, it has the problem of low computational efficiency. The research content of this paper includes the detection of the main equipment in marine engine rooms. Due to the large amount of equipment in the engine room, there will be mutual occlusion, affecting the detection effect. This paper adopts Gaussian weighted soft-NMS instead of weighted NMS as the NMS method of YOLOv5, and its calculation formula is as follows:

(9) $$s_{i}=s_{i}e^{\frac{iou{\left(M,b_{i}\right)}^{2}}{\sigma }}$$

Soft-NMS has the advantages of easy implementation and high efficiency. Soft-NMS is proposed to deal with the problem of mutual occlusion of target boxes. The screening process will not directly delete the candidate boxes whose IOU value is greater than the threshold, but attenuates their confidence. The larger the IOU value of the two boxes, the faster the confidence decays, and the faster the score of the corresponding box decreases. Finally, the corresponding candidate boxes are obtained through IOU threshold screening [31]. When selecting candidate boxes, soft-NMS uses a relatively soft method, which has a good solution to the problem of false target detection and missing detection.

#### 3.2.4. Hard-Swish Activation Function

**Figure 12:**
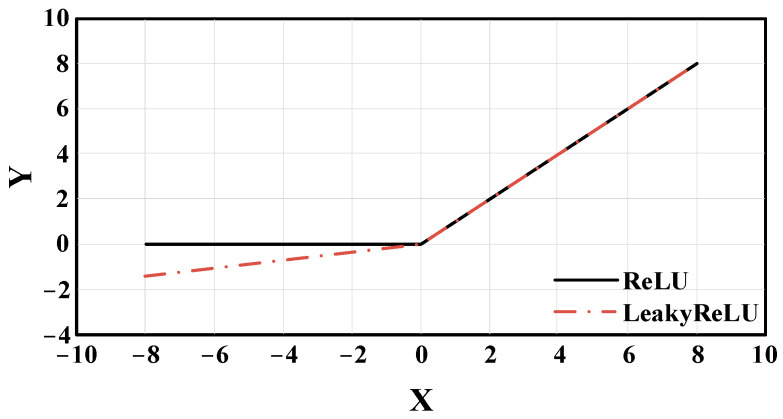
Graph of LeakyReLU and ReLU activation function.

The activation function of YOLOv5 is LeakyReLU, which evolved based on the rectified linear unit (ReLU) activation function. Figure 12 shows their function curve. Compared with ReLU, LeakyReLU has a better effect on negative values, and the overall function interval is not zero. It solves the problem that some network parameters cannot be updated. While LeakyReLU is better, in theory, it is less effective in practice.

**Figure 13:**
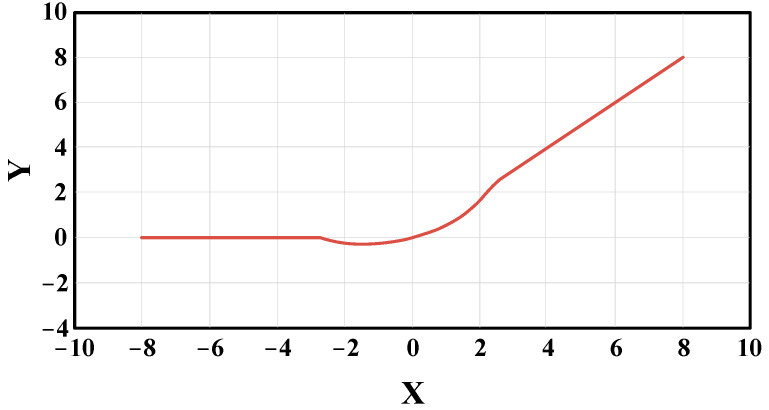
Graph of hard-swish activation function.

In this paper, hard-swish is used to replace the LeakyReLU as the activation function of YOLOv5. The curve of the hard-swish activation function is shown in Figure 13. Compared with the LeakyReLU activation function, hard-swish has the advantages of lower computational cost and higher stability. At the same time, the curve of the hard-swish activation function is smoother at the zero point, and its gradient descent effect is better than that of the LeakyReLU activation function. The hard-swish activation function can be calculated as follows:

(10) $$hard-swish(x)=\left\{ \begin{array}{l} 0,\;x\leq -3\;\\ \frac{x(x+3)}{6},\;otherwise\\ x,\;x\geq 3 \end{array}\right.$$

## 4. Experiments

This section verifies the validity of the detection model on the MEMER dataset. Firstly, we introduce the building process of the dataset, including image collection, data annotation, data augmentation, dataset construction, and analysis in Section 4.1. Next, the experimental environment, hardware and software configurations are introduced in Section 4.2. Then, the performance evaluation indicators commonly used in object detection are introduced in Section 4.3. Finally, the effectiveness of the model is verified in Section 4.4. through the PASCAL VOC 2007 test set and the MEMER test set. The superiority of the improved YOLOv5 model is verified by a visual comparison of some detection results.

### 4.1. MEMER Dataset

**Figure 14:**
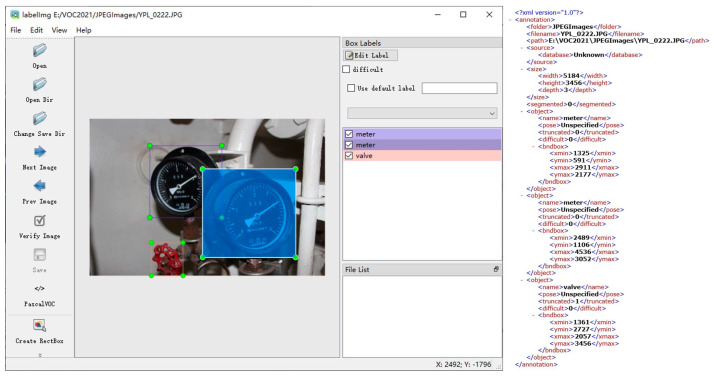
Example of LabelImg interface and XML.

The original photos used in the dataset were taken by our virtual marine engine rooms team at Dalian Maritime University. A Canon EOS 700D digital camera shot 1475 photos with a resolution of 5184 × 3456. Raw image samples cover a variety of highly complex situations. The primary workload of dataset construction is image annotation. After the file name serialization and file format unification of the original image, as shown in Figure 14, LabelImg (an annotation tool) is used to annotate all the equipment.

**Figure 15:**
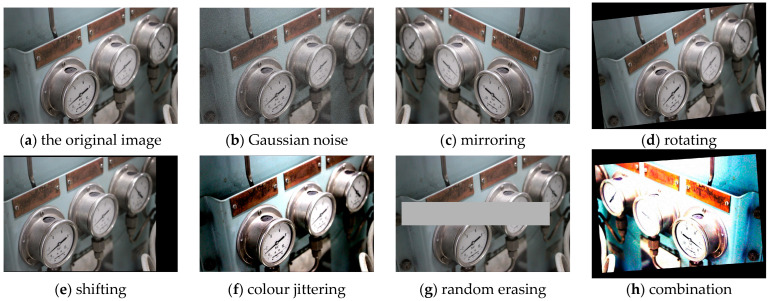
Examples of data augmentation.

Due to the limited number of the samples, such as the main engine, we adopt a combination of data augmentation methods to enhance the robustness of the model, including Gaussian noise, mirroring, rotating, shifting, colour jittering and random erasing [32]. Some examples after data augmentation are shown in Figure 15. Then, the number of samples is expanded from the original 1475 to 7375. Finally, augmented images are randomly divided according to the ratio of 7:2:1, training, validation, and test set, respectively. So far, the construction of the MEMER dataset has been completed.

### 4.2. Configurations and Situation

The configuration and details of the experimental verification platform are shown in Table 1. Among them, the operating system is Windows 10, the graphics processing unit (GPU) is NVIDIA GeForce GTX 1660Ti, and the central processing unit (CPU) is Intel I7-9700 with eight cores, random access memory (RAM) is 16 GB, and the code integration development environment (IDE) is PyCharm. The detection model is built based on the programming language Python 3.7.0 and the DL framework GPU-based PyTorch 1.4.0. Finally, training acceleration, reasoning test and verification are completed in CUDA 11.3.

### 4.3. Criteria

In the field of object detection, the mean average precision (mAP) index is usually used to evaluate the model performance. The definition of mAP is related to the recall and accuracy rate. In terms of original samples, the recall rate refers to the proportion of the number of correctly predicted samples of a certain type of equipment in the number of actual samples of this type of equipment. It can judge the degree of missed detection of the model. The larger the recall rate is, the less the missed detection will be. In terms of prediction results, accuracy refers to the proportion of the number of correct samples predicted for a certain type of equipment in all samples whose prediction results are the type of equipment. It can judge the degree of false detection of the model. The greater the accuracy is, the smaller the false detection rate will be. In order to better define the recall rate and accuracy, we established the following definitions: the true positive example TP refers to the original sample as a positive sample and the model prediction result as a positive sample; the false positive example FP means that the original sample is negative and the model prediction result is positive. The true negative example TN refers to the original sample as negative and the model prediction as negative; the false negative example FN means that the original sample is positive and the model prediction is negative [33].

According to the above definition, TP+FP+TN+FN is the number of total samples, TP+FN represents the number of actual positive samples, TP+FP represents the number of positive samples predicted, FP+TN represents the number of actual negative samples and TN+FN represents the number of negative samples predicted. According to the definition, the calculation formula of recall rate and accuracy is as follows:

(11) $$\mathrm{Recall}=\frac{\mathrm{TP}}{\mathrm{TP}+\mathrm{FN}}\times 100\%$$

(12) $$\mathrm{Precision}=\frac{\mathrm{TP}}{\mathrm{TP}+\mathrm{FP}}\times 100\%$$

If the recall rate (recall) is set as the horizontal axis, the accuracy rate (precision) is set as the vertical axis, and the recall–precision curve can reflect the recognition accuracy and coverage ability of the classifier for positive samples. The area under the curve is the current average precision (AP). In the case of multi-category targets, the mAP index is involved, which is used to calculate the average value of all categories of AP. The formula is as follows:

(13) $$\mathrm{AP}=\int_{0}^{1}p(r)dr$$

(14) $$\mathrm{mAP}=\frac{\sum_{i=1}^{C}\mathrm{AP}(C_{i})}{C}$$

The p(r) represents the recalling–precision curve mapping, $C_{i}$ is the i^th^ category and C is the total number of categories.

Besides mAP, detection time (time) and frames per second (FPS) are also used to measure model efficiency in this paper. Time represents the average time required by the model to detect the target area in the image. The larger the time is, the worse the performance will be. On the contrary, the smaller the FPS is, the slower the model checking speed will be.

### 4.4. Verification

For model training, this paper first obtains the open-source weight file released by YOLOv5 and uses its corresponding training model for pre-training. Through transfer learning, the PASCAL VOC 2007 + 2012 training set is used to train and fine-tune the model. Then, the trained weight file of PASCAL VOC is used as the pre-training model for the improved YOLOv5, and the MEMER training set is used to continue to train the model. When training on PASCAL VOC, the batch size is set as 8, and the number of iterations and the initial learning rate is set as 100 epochs and 1 × 10^−3^, respectively. When the number of iterations reaches the 50th and 80th epoch, the learning rate decreases to 10% of the previous learning rate, respectively. As for the training on MEMER, the batch size is still set as 8, the number of iterations is set as 80 epochs, and the initial learning rate is set as 1 × 10^−4^. If the model’s total loss does not evidently decrease in four consecutive epochs, the learning rate will decrease to half of the previous rate.

#### 4.4.1. Comparison on PASCAL VOC

In order to evaluate the proposed detection model for the main equipment in marine engine rooms, this section compares the model with the PASCAL VOC dataset from the World Class Computer Vision Challenge. Firstly, the baseline and improved model were trained on the PASCAL VOC 2007 + 2012 training set, then tested and compared with the PASCAL VOC 2007 test set. In addition, under the same setting, as quoted in reference [34], the result is compared with the current mainstream detection model Faster R-CNN, YOLOv3, SSD, DSSD, RSSD, FSSD and RetinaNet, which fully demonstrates the advantages of the proposed model. The specific comparative experiment results are shown in Table 2.

According to Table 2, when the resolution of the input image is 640 × 640, the mAP of the model in this paper can reach 79.9%, and the detection speed is 22.6 FPS. Compared with the two-stage target detection algorithm Faster R-CNN, the mAP is improved by 9.2/4.6% and the FPS is improved by 15.6/20.2. Compared with the other target detection algorithms YOLOv3, SSD, DSSD, RSSD, FSSD and RetinaNet, the mAP of the proposed model is improved by 4.8%, 3.5%, 1.7%, 1.8%, 1.4% and 0.8%, respectively. Compared with the RetinaNet with the same input size, the mAP is only improved by 0.5%, but the FPS is increased by 23.5% from 18.3 to 22.6. The improvement is significant, which proves that the improved model has certain superiority.

#### 4.4.2. Validations Using MEMER

The ablation experiment is carried out on the MEMER test set to validate the efficacy of each improvement, including the CIoU_Loss loss function, soft-NMS, and hard-swish activation function. By comparing the difference of mAP and time, the performance of each improved strategy is presented in Table 3. It can be observed that when each strategy is used separately, the CIoU_Loss loss function improves the performance the best by 3.67% on mAP. Although the hard-swish activation function demonstrates the lowest efficacy in terms of model performance promotion when applied on the model alone, the effect, especially regarding the detection time, is more significant when based on other measures than when it is applied alone.

The comparison between models training with and without data augmentation is performed to verify the positive effect of data augmentation measures on model performance promotion, and the results are shown in the following Table 4. The M1 represents the model trained with the original dataset. The M2 represents the model trained with the augmented dataset. The D1 represents the test set of the original dataset. The D2 represents the test set of the augmented dataset. The D3 can be viewed as a test set collected from the engine room of a new ship type, which is outside this dataset. As can be observed from the table, the generalization ability and robustness of the model trained by the augmented dataset have been significantly improved.

The improved YOLOv5 model is compared and analyzed with the current mainstream detection model on the MEMER test set. Table 5 shows the AP value, mAP value and FPS of each model for the seven types of main equipment targets in the MEMER test set.

As observed in Table 5, the improved model proposed in this paper is higher than others in mAP. Specifically, it is 10.42%, 10.31%, 6.33%, 7.83%, 5.96% higher than Faster R-CNN (76.13%), SSD (76.21%), RSSD (79.06%), FSSD (77.96%), RetinaNet (79.34%), respectively. It is also 6.54% higher than the baseline YOLOv5 model. It can be observed that the improved model has great advantages in detection accuracy and recognition speed.

**Figure 16:**
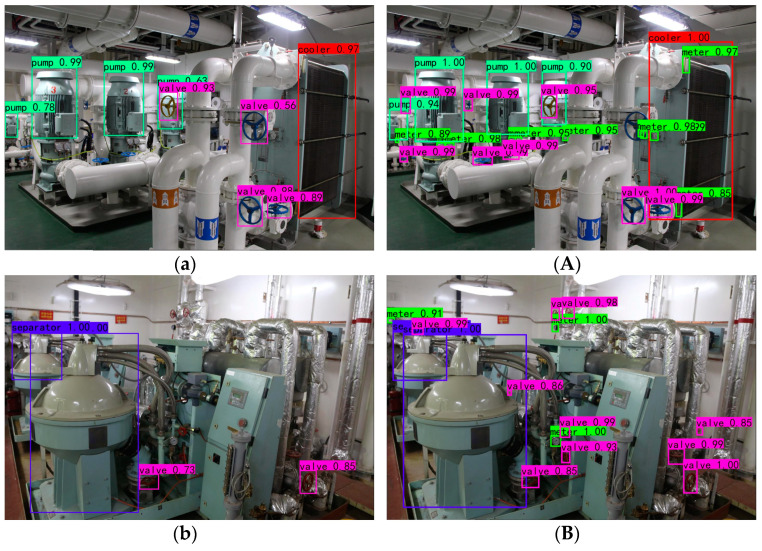

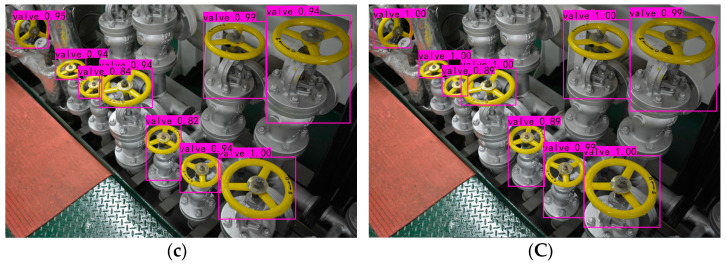
Detection results visualization in actual scenes. The images of (**a**–**c**) are detection results of baseline YOLOv5, while (**A**–**C**) are detection results of improved YOLOv5.

Typical images in the MEMER test set are selected for comparison to visually demonstrate the effect of the improved YOLOv5 model on main equipment detection in actual marine engine room scenes, and the results are shown in Figure 16. Among them, the left side of the display is the baseline model recognition result, and the improved model recognition result is on the right. The contrast results show that the improved model is not only improved with regard to single equipment identification precision, but it also can identify additional small targets, such as valves. The improved model effectively improves the missing detection phenomenon of small target equipment in scenes with large-scale differences and improves the robustness of the model in single-class multi-target detection.

## 5. Conclusions and Discussions

This paper proposes a recognition model for the main equipment in marine engine rooms based on improved YOLOv5 and mainly carries the following tasks: constructs the MEMER dataset, builds a model and carries out optimization design and verifies the model by comparison. In order to reduce the cost of model training and improve the speed of recognition, the channel pruning based on BN layer weight value is firstly carried out. In order to improve the equipment identification accuracy in the complex environment of engine rooms, the CIoU_Loss loss function and hard-swish activation function are used to improve the baseline algorithm, and soft-NMS is used as the non-maximum suppression method to reduce the false detection rate and missed detection rate. Finally, the experiment proves the superiority of the improved model.

The YOLOv5 model proposed in this paper can realize the appearance of the main equipment identification but still has the following disadvantages: (1) the lack of equipment category and quantity in the MEMER data set. The current dataset consists of diesel engines, pumps, coolers, separators, valves meters and reservoirs, but the actual marine engine room contains much more equipment. In the future, more abundant photos of marine engine room equipment should be collected to improve the data set, and a more advanced study on its appearance should be carried out. (2) The detection accuracy of meters and reservoirs is still low, and their AP has not reached 70%. In the process of training, we tried to modify the parameters and framework, but the results are still not ideal. Therefore, the results of mAP are mainly considered, and the research of meters and reservoirs will be further explored in the future.

With the continuous improvement of intelligent ship laws and regulations, computer vision and other advanced technologies will be more and more applied in the field of marine and ocean engineering. For example, visual technology assists PSC prosecutors in ship inspection, and unmanned vessels assist rescuers in maritime search and rescue, etc.

## Figures and Tables

**Table 1:** The configuration of experimental platform.

Configuration	Detail
Operating System	Windows 10
GPU	NVIDIA GeForce GTX1660Ti
CPU	Inter i7-9700 (3.00 GHz) 8-core
RAM	16 GB
IDE	PyCharm 2020.1.4
Framework	GPU-based PyTorch-1.4.0
Toolkit	CUDA 11.3

**Table 2:** Comparison of the test detection results on the PASCAL VOC 2007.

Model	Pre-Train	Input Size	GPU	FPS	mAP (%)
Faster R-CNN	√	600 × 1000	Titan X	7	73.2
Faster R-CNN	√	600 × 1000	K40	2.4	76.4
YOLOv3	√	352 × 352	Titan X	19.9	75.7
SSD	√	300 × 300	Titan X	46	77.2
DSSD	√	321 × 321	Titan X	9.5	78.6
RSSD	√	300 × 300	Titan X	35	78.5
FSSD	√	300 × 300	1080Ti	65.8	78.8
RetinaNet	√	600 × 600	1660Ti	17.4	79.3
YOLOv5	√	640 × 640	1660Ti	18.3	79.5
Improved YOLOv5	√	640 × 640	1660Ti	22.6	79.9

**Table 3:** Comparison of different improvement measures.

Model	CIoU_Loss	Soft-NMS	Hard-Swish	Time (ms)	mAP (%)
Baseline	-	-	-	56	78.91
Schemes	√	-	-	60	82.58
	-	√	-	57	80.23
	-	-	√	49	79.35
	-	√	√	56	80.44
	√	-	√	57	82.36
	√	√	-	62	83.62
	√	√	√	52	84.07

**Table 4:** Comparison between models with and without using data augmentation.

Model	mAP (%)		
	D1	D2	D3
M1	84.2	81.3	78.6
M2	84.8	84.7	84.2

**Table 5:** Comparison of the detection results on the MEMER test set.

Model	AP(%)							FPS	mAP (%)
	Engine	Pump	Cooler	Separator	Meter	Reservoir	Valve		
Faster R-CNN	93.77	82.11	90.96	84.83	43.81	86.95	50.49	8.53	76.13
SSD	100	89.46	83.53	91.71	46.22	71.05	51.48	27.99	76.21
RSSD	100	90.39	85.85	93.90	49.53	78.57	55.18	17.94	79.06
FSSD	100	89.79	84.30	93.60	47.94	76.49	53.62	24.26	77.96
RetinaNet	100	94.03	93.91	95.69	57.21	48.81	70.86	17.24	79.34
YOLOv5	100	94.52	94.02	94.97	55.02	53.35	58.54	19.05	78.91
Improved YOLOv5	100	95.91	94.29	98.54	64.21	60.23	75.32	25.07	84.07

## Data Availability

The processed data cannot be shared at this time, as the data also form part of an ongoing study.
